# The Prevalence of Congenital Color Vision Abnormality Among Patients Attending a Tertiary Eye Care Center in Southern India

**DOI:** 10.7759/cureus.43837

**Published:** 2023-08-21

**Authors:** Satyasri B, Praveen Kumar K V

**Affiliations:** 1 Ophthalmology, Sri Lakshmi Narayana Institute of Medical Sciences, Puducherry, IND; 2 Ophthalmology, ACSR Government Medical College, Nellore, IND

**Keywords:** protanopia, tritanopia, deuteronopia, hereditary eye disorders, colour vision

## Abstract

Background and objective

Color vision abnormality is a condition where the faculty to identify one or more primary colors is either defective (anomalous) or absent (anopia). Occupations like armed forces, merchant navy, navigation, and police and fire services require normal color vision. There is a scarcity of data in the literature regarding the prevalence of congenital color vision abnormality in patients in South India. In light of this, the present study aimed to find the prevalence of congenital color vision abnormality in patients attending the Outpatient Department (OPD) at a tertiary eye care center in South India.

Materials and methods

This was a descriptive cross-sectional study conducted at a tertiary eye care center in South India from December 2014 to December 2016. Patients with a best-corrected visual acuity of 20/20, normal direct and consensual pupillary light reflex, normal anterior segment, and undilated fundus examination were included. Color vision was assessed using Ishihara pseudoisochromatic plates, American Optical Hardy-Rand-Rittler (AO HRR), and then the Farnsworth Munsell D15 arrangement test. All the results were tabulated and statistically analyzed. Statistical significance was calculated using the ANOVA test.

Results

A total of 371 patients were screened for color vision abnormality; 184 (49.59%) patients were males and 187 (50.40%) were females. Out of 371 patients in the study, 363 (98.10%) had normal color vision while eight patients (2.16%) had color vision abnormality. Of the eight patients with color vision abnormalities, six (75%) had abnormal color vision on all three tests and two (25%) had an abnormality on only two tests (Ishihara and AO HRR). Out of 184 males in the study, eight patients (4.34%) had abnormal color vision while none of the 187 females had color vision abnormality; this difference was statistically significant (p=0.03).

Conclusions

Early diagnosis and providing education and awareness of this condition are necessary as part of guiding young people with regard to their career options and we recommend that all children should undergo color vision screening before entering high school.

## Introduction

Color vision refers to the ability to discriminate a light stimulus as a function of its wavelength, and the ability to see color distinguishes humans from other species [1]. Color vision abnormality is a condition in which the faculty to identify one or more primary colors is either defective (anomalous) or absent (anopia). The prevalence of color vision abnormality has been found to vary between different races, tribes, and ethnic groups. Among the congenital color vision defects, the X-linked recessive disorder is the most common variant and results in difficulty in distinguishing colors in the red-green spectrum.

Acquired color vision deficiency can be due to ocular causes like macular degeneration and glaucoma. In a study conducted in Nepal, the prevalence of congenital color vision deficiency in the general population was 8% in males and 0.5% in females [2,3]. Screening for color vision abnormality is essential for a wide variety of purposes, including obtaining entry into certain professions. Occupations like armed forces, merchant navy, navigation, and police and fire services require normal color vision.

Screening of the population for early identification of children with color vision abnormalities is widely practiced in various industrialized countries, which aids in guiding them early about the future occupations for which normal color vision is required [4-5]. Screening for color vision abnormality can also be helpful in recognizing congenital and acquired disorders, classification of acquired disorders in patients with various ocular diseases, assessment of response to treatment, and tracking recovery from an ocular disease or trauma.

There is scant data in the literature about the prevalence of congenital color vision abnormality in patients in South India; hence, the present study was conducted to examine the prevalence of congenital color vision abnormality among the patients attending the outpatient department (OPD) of a tertiary eye care center in South India.

## Materials and methods

This was a descriptive cross-sectional study conducted among the patients attending the OPD of a tertiary eye care center in South India from December 2014 to December 2016. Informed consent was obtained from all the participants in the study. Ethical committee approval was also obtained. Patients with a best-corrected visual acuity of 20/20, normal direct and consensual pupillary light reflex, no relative afferent pupillary defect (RAPD), unremarkable slit-lamp biomicroscopic, and normal undilated fundus examination were included in the study. Patients in the nonverbal age group, Illiterate patients, patients unable to understand the nature of the test, patients with a previous history of any ocular disease, previous ocular surgeries, ocular trauma, high myopia, lenticular changes, and patients on chronic drug therapy for more than a month were excluded from the study. A multistage sampling method was employed.

Demographic data including age, gender, occupation, history of any eye disorder, head trauma or ocular trauma, medical history, and awareness about color vision abnormality among themselves or their family members were inquired. Patients attending the OPD for regular checkups with no ocular complaints like pain, redness, and photophobia were included in the study. Among them, patients who were able to read the Snellen visual acuity chart were selected. Thorough slit-lamp examination and undilated fundus examination were performed among the selected subjects. Visual field testing by confrontation was also done in the selected subjects. In the selected subjects, color vision was assessed using Ishihara pseudoisochromatic plates, American Optical Hardy-Rand-Rittler (AO HRR), and then the Farnsworth Munsell D15 arrangement test. The tests were done separately for each eye of each patient, i.e., monocular testing was carried out. For all individuals with color vision abnormality on any of the three tests, dilated fundus examination was performed with an indirect ophthalmoscope and slit-lamp biomicroscopy using a 90 D lens to rule out any missed ocular pathology.

**Figure 1:**
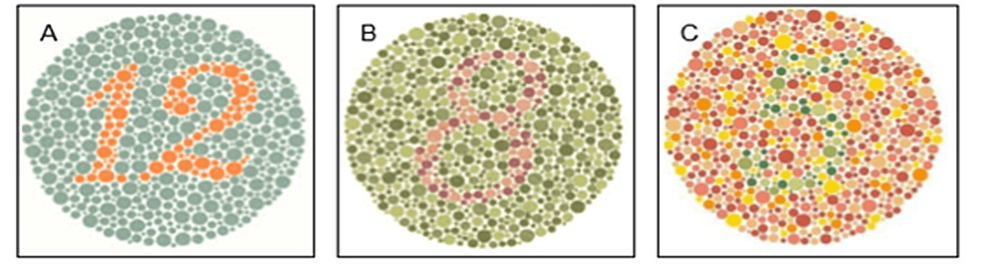
Figure showing the demonstration (A), transformation (B), and vanishing (C) design plates on the Ishihara chart

While testing with Ishihara charts, the testing plates are held at 75 cm from the eye and tilted at a right angle to the line of vision. The test is carried out in a properly lighted place. The patient is asked to read out the numbers on the plates. The time allowed for reading the number on a plate should not exceed three seconds. The patients are tested monocularly. Patients with differences between both eyes are excluded. The responses are noted on the response collection forms. Assessment of the reading of the plate determines the normality or abnormality of color vision and also the type of color vision abnormality. No record sheet is provided, but scoring instructions accompanies each test. The demonstration plate is included in the score. In the 38-plate edition, four errors or fewer are considered normal; eight errors or more are regarded as abnormal and these patients are considered to have red-green color vision abnormality. Further classification into protan and deutan types is based on the ability to read plates 22, 23, 24, and 25. Figure 1 shows the various design plates on the Ishihara chart.

In AO HRR pseudoisochromatic plates, the test consists of 24 plates in which colored symbols of various sizes and lightness form outlines of symbols on a background of gray circles of various sizes and lightness. These symbols are a cross, a circle, and a triangle. The first four plates are demonstration plates: three have symbols and one is blank. These four plates are intended to screen for hysteria and malingering. Observers with visual acuity better than 20/200 should be able to give correct answers. The demonstration plates are followed by six screening plates (two for blue-yellow and four for red-green color vision abnormality). The plates are of the vanishing type, and the hues in the symbols for the screening plates are close to gray. The screening series is followed by 14 hidden-figure diagnostic plates. Ten of these are for red-green color abnormalities. Four of the diagnostic plates are for blue-yellow color vision abnormalities. Patients are made to identify the symbols and the responses are noted on the response collection form. A patient who gives the correct response to all six screening plates is adjudged to have normal color vision. A patient who makes one or more errors in the screening plates but none in the subsequent diagnostic plates, and gives correct responses to all the screening plates upon retesting (5-10), is considered to have normal color vision. A patient is protan if the total number of checks in the protan column is greater than in the deutan column, and deutan if the total number of checks in the deutan column is greater than in the protan column; the patient is unclassified in terms of red-green color vision abnormality if the number of checks is same in both columns or if errors have been made only in the screening plates. A patient is tritan if the total number of checks in the tritan column is greater than the tetaran column, and tetaran if the total number of checks in the tetaran column is greater than in the tritan column. The patient is unclassified with regard to blue-yellow color vision abnormality if the number of checks is the same in both columns or if errors have been made only in the screening plates.

The last group of plates in which error occurs points to the extent of the patient’s color vision abnormality. For example, in the case of red-green color vision abnormality, if the last error occurs in either group of plates 7-10 or 11-15 and no error occurs in plates 16-20, the abnormality is mild in extent. If the last error is in 16-18 and no error occurs in 19-20, the abnormality is medium in extent, and if the error occurs in plates 19-20, the defect is strong. The same logic applies to blue-yellow color vision abnormality. Figures 2-4 show various plates in the HRR test.

**Figure 2:**
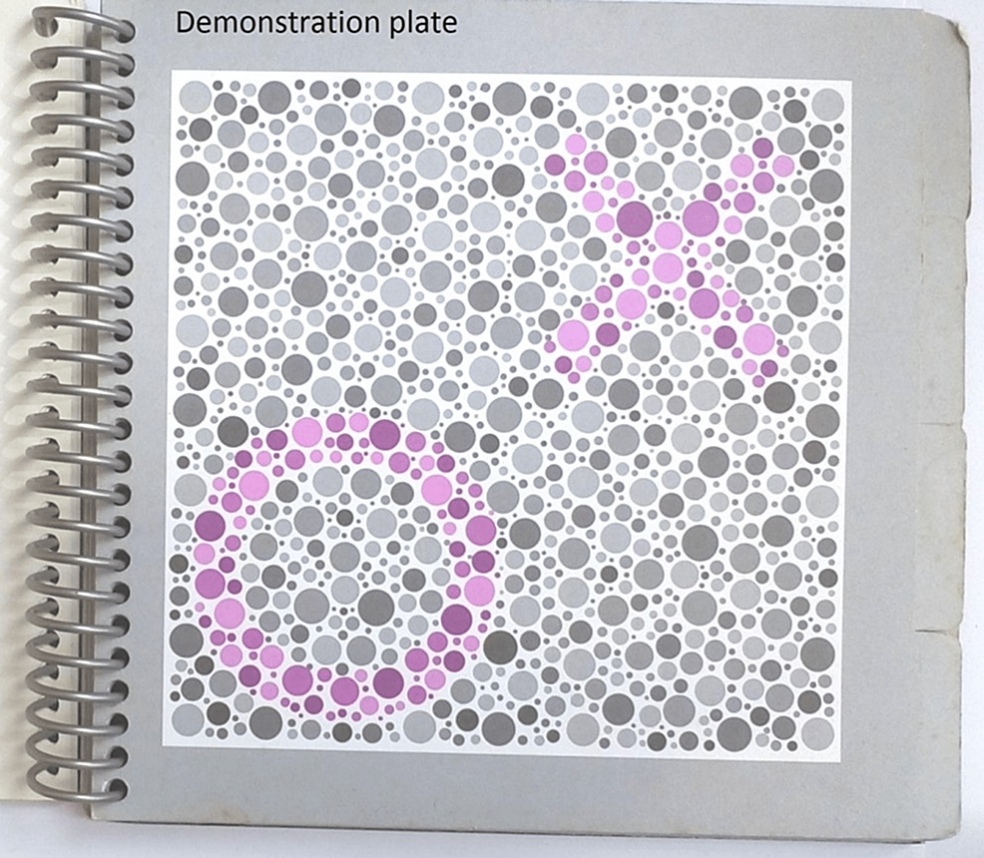
Figure showing the demonstration plate on the HRR test HRR: Hardy-Rand-Rittler

**Figure 3:**
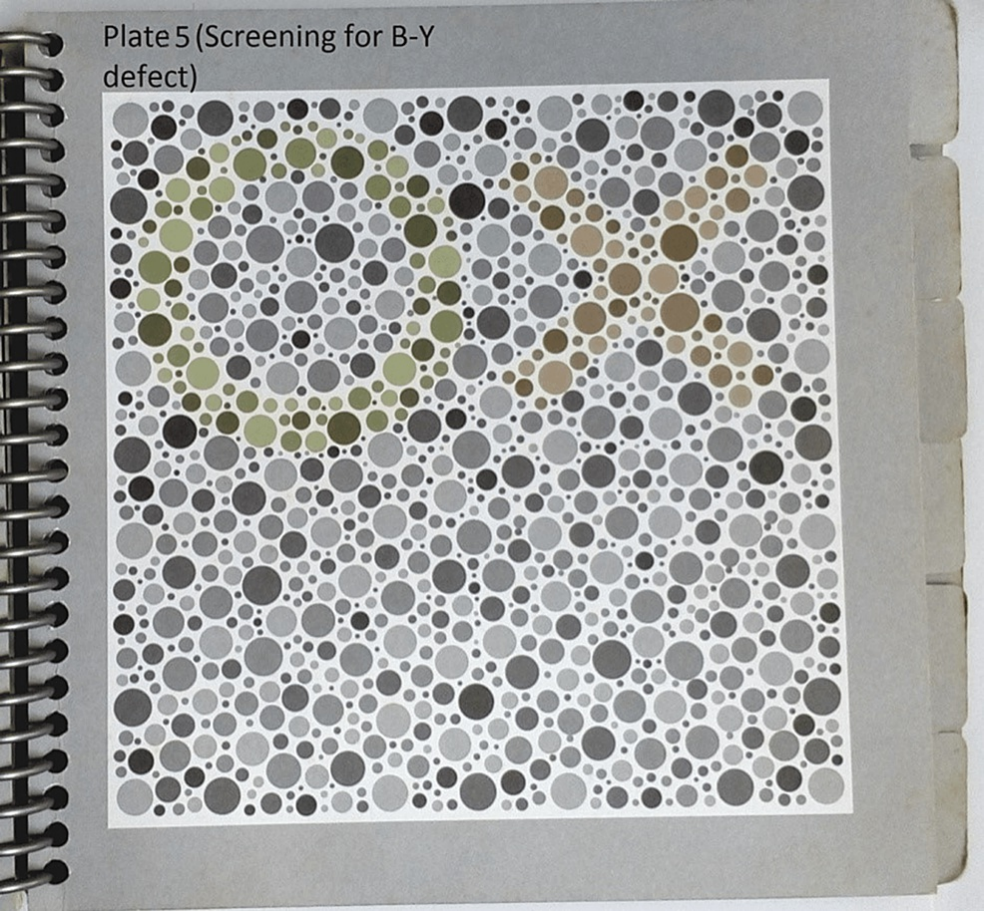
Figure showing HRR plate for screening for blue-yellow deficiency HRR: Hardy-Rand-Rittler

**Figure 4:**
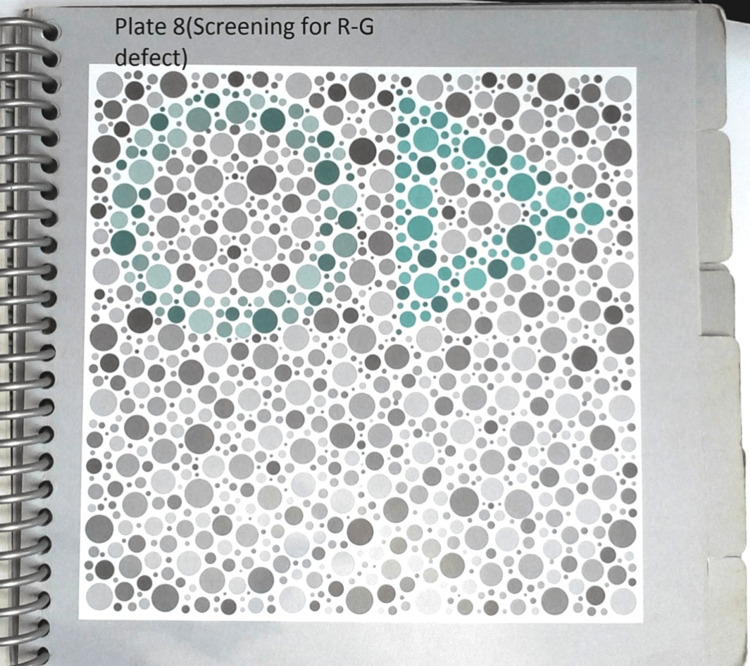
Figure showing red-green deficiency screening plate on the HRR test HRR: Hardy-Rand-Rittler

In the Farnsworth Munsell 100 Hue test, the test is conducted at a distance of 50 cm against a black background. It consists of 16 colored discs. One of them is a reference cap, which is fixed. The patient is made to wear gloves and instructed to select the color disc, which closely matches the reference cap, and then asked to place it into the box and slide it next to the reference cap. The patient is made to continue to select the next closest color disc and place each in sequence. All 15 discs are arranged by the patient in a sequence. The patient is permitted to alter the sequence prior to completion if he/she wishes to do so. However, the total time should not exceed two minutes. Scoring is done by reading the numbers on the bottom of each color disc. The sequence is recorded on the respective response collection form and assessed for any abnormality according to the instruction guide provided with the test. The order of the caps is plotted directly on the score sheet on a diagram that shows correct cap positions extending in a circle from the reference cap. In correct order, the lines retrace the hue circle. Errors occur when caps are misplaced and not in the correct order. Subjects with normal color vision will make at most only one or two minor errors.

**Figure 5:**
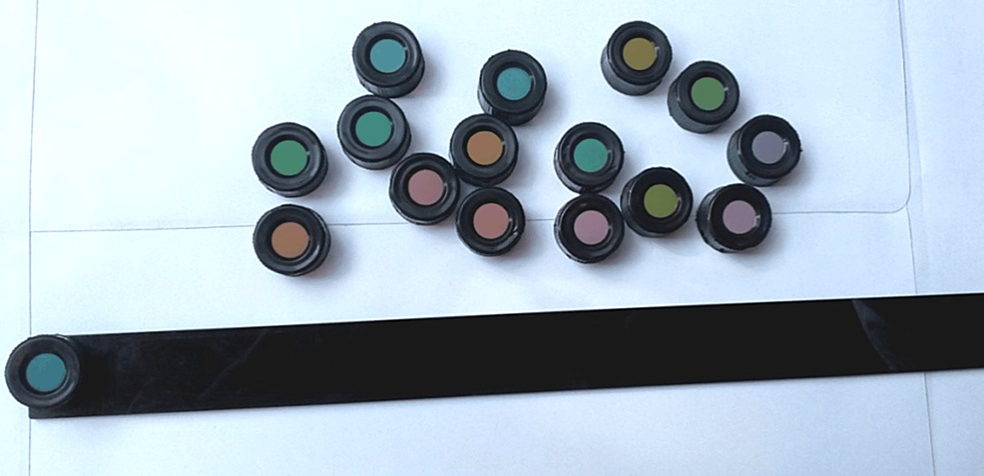
Figure showing colored discs of the Farnsworth Munsell 100 Hue test

According to the original design, the test is considered abnormal if an observer makes two or more major crossovers that are parallel to any axis line on the scoring sheet. Dichromats and extreme anomalous trichromats make multiple (612) crossovers, forming a nearly parallel series of lines. The axis of the crossover lines is characteristic of the type of defect; the axes corresponding to protan, deutan, and tritan abnormalities are indicated on the scoring sheet. The result is interpreted based on the axis line on the score sheet. Figure 5 shows colored discs of the Farnsworth Munsell 100 Hue test.

All the results were tabulated and statistically analyzed. The results were expressed in percentages, mean, standard deviation (SD), and coefficient of variation (CV). Statistical significance was calculated by using the ANOVA test. The variations in quantitative performance and for testing the hypothesis were measured with the help of ANOVA. The confidence interval for the percentage was calculated using the Wilson method.

## Results

A total of 371 patients were screened for color vision abnormality. More than 80% of the patients in our study were in the age group of 10-30 years (Table 1).

**Table 1:** Age distribution of the patients in the study

S. no	Age group, years	N	%
1	5-9	1	0.2%
2	10-19	131	35.3%
3	20-29	176	47.50%
4	30-39	56	15.0%
5	40-45	7	1.9%
6	Total	371	100%

Out of 371 patients in the study, 184 (49.59%) were males and 187 (50.40%) were females (Table 2).

**Table 2:** Gender distribution of the patients in the study

S. no.	Gender	N	%
1	Males	184	49.59%
2	Females	187	50.40%
3	Total	371	

With respect to the refractive status, 167 patients (45%) were emmetropic, 159 patients (43%) were myopic (including myopic astigmatism), and 45 patients (12%) were hypermetropic (including hypermetropic astigmatism) (Table 3).

**Table 3:** Refractive status of the patients in the study

S. no.	Refractive status	N	%
1	Emmetropia	167	45%
2	Myopia (including myopic astigmatism)	159	43%
3	Hypermetropia (including hypermetropic astigmatism)	45	12%
4	Total	371	100%

Out of 371 patients in the study, 363 patients (98.10%) had normal color vision, and eight patients (2.16%) had color vision abnormalities. Out of the eight patients with color vision abnormalities, six patients (75%) had an abnormal color vision on all three tests, and two patients (25%) had an abnormality on only two tests (Ishihara and AO HRR). Two (25%) out of eight patients with color vision abnormality on Ishihara and AO HRR arranged the discs of the Farnsworth Munsell D15 test in an order that could not be classified into any of the abnormalities. These two patients had unclassified red-green color vision abnormality on Ishihara pseudoisochromatic plates and mild unclassified red-green color vision abnormality on the AO HRR pseudoisochromatic plates test (Table 4).

**Table 4:** Number of tests on which patients had color vision abnormality

S. no.	Number of tests	N	%
1	Abnormality on all three tests	6	75%
2	Abnormality on two tests	2	25%
3	Abnormality on one test	0	0%
4	Total	8	100%

Out of 184 males in the study, eight patients (4.34%) had abnormal color vision while none of the 187 females had color vision abnormality; this difference was statistically significant (p=0.03). All eight patients with color vision abnormalities had red-green color vision abnormalities and none had blue-yellow abnormalities. The red-green color vision abnormalities seen in these patients were unclassified red-green abnormality in two patients (0.5%), while three patients had deutan abnormality, of which two were deuteranomaly (0.54%), and one was deuteranopia (0.26%). Three patients had protan abnormality, of which two were protanomaly (0.54%) and one (0.26%) was protanopia (Table 5).

**Table 5:** Prevalence of the various types of color vision abnormality in the study

S. no.	Type of color vision abnormality	Subtype			N	%
1	Red-green	Unclassified red-green abnormality			2	0.5%
		Duetan		Deuteranomaly (mild-medium deutan)	2	0.54%
				Deuteranopia (strong deutan)	1	0.26%
		Protan		Protanomaly (mild-medium protan)	2	0.54%
				Protanopia (strong protan)	1	0.26%
2	Blue-yellow				0	0%
3	Total				8	2.16%

Out of eight patients with color vision abnormality, six patients (75%) were aware of their defects before presenting to the hospital and two patients (25%) were unaware and were detected on our screening. Out of six patients who were aware of their defect, four patients (50%) were identified to have defects elsewhere on testing for their occupational purposes and two patients (25%) were identified by their peers based on their inappropriate identification of colors.

Out of eight patients with color vision abnormalities, five patients (62.5%) were emmetropes, three patients were myopes including myopic astigmatism (37.5%), and none of the patients had hypermetropia; this difference was not statistically significant.

## Discussion

Color vision abnormality is a condition in which the faculty to perceive one or more primary colors is either defective or absent. Congenital color vision abnormality can be classified based on the cone affected as anomalous trichromacy, dichromacy, and monochromacy. Tritan abnormalities are rare, whereas protan and deutan abnormalities are relatively common. Protan and deutan abnormalities are inherited as X-linked recessive characteristics and hence are more common in males. Acquired color vision abnormality develops later in life and can affect men and women equally. Diseases that damage the optic nerve or the retina of the eye can cause acquired color vision abnormality. Being a genetic disorder, the prevalence of color vision abnormality has been found to vary between different races, tribes, and ethnic groups. No major studies have been conducted so far to assess the prevalence of congenital color vision abnormality in Southern India and hence this study was planned with a view to examine the prevalence of congenital color vision abnormalities at a tertiary center in South India.

A total of 371 patients were screened for color vision abnormality. More than 80% of the patients in our study were in the age group of 10-30 years. Very few of the patients in the study were aged above 40 years and below 10 years as most patients above the age of 40 years may have lenticular changes that might affect color vision. Patients below the age of 10 years may not understand and perform the test appropriately.

In the present study, patients were screened for color vision abnormality using Ishihara pseudoisochromatic plate tests, AO HRR pseudoisochromatic plate test, and the Farnsworth Munsell D-15 test. Ishihara pseudoisochromatic plates test is the most commonly used test while screening for occupational purposes as well as the most commonly available test in a clinical setting, and hence the test was used. But this test does not grade the severity of the defect and also cannot detect tritan abnormality. AO HRR pseudoisochromatic plates can differentiate between protan abnormality and deutan abnormality, grade the severity of the defect as mild, moderate, and severe, and can also test for tritan abnormality. Hence, AO HRR pseudoisochromatic plates were used. Farnsworth Munsell D-15 arrangement test was included as an additional test as it was available in our setting. Out of the three tests, Ishihara and HRR tests are pseudoisochromatic tests and D 15 is an arrangement test.

Eight out of 371 patients in our study were found to have color vision abnormalities. The prevalence of congenital color vision abnormality in the general population in our study was 2.15%. The prevalence of color vision abnormality in the general population in our study was comparable to other studies. The prevalence in our study was in accordance with the study conducted by Rahman et al., where the incidence of color blindness was found to be 2.209% in the Libyan population. The prevalence of color vision abnormality in our study was less than the prevalence in the studies from Australia, the USA, North Africa, and China [6-9]. All the subjects who were tested to have color vision abnormality were in the age group of 20-25 years. This might be attributed to the fact that 80% of the patients in our study were in the age group of 10-30 years.

The patients who were found to have color vision abnormalities were all males. None of the females in the study had color vision abnormalities. The prevalence of congenital color vision abnormality among males in our study was 4.34%. The prevalence of color vision abnormality in males in a study from Saudi Arabia was less than the prevalence of color vision abnormality among males in our study [10]. The prevalence of color vision abnormality in males in our study was in agreement with various other studies conducted in India, except for Muslim males in Manipur [11-14]. Since it is inherited as an X-linked recessive pattern, color vision abnormality is more common in males and rare in females. A study by Rahman et al. found the prevalence of color vision abnormality in females to be 0.84%, whereas Shresta et al. reported that none of the females in their study were found to have color vision abnormality, which is in line with our study [14].

In the present study, eight patients had red-green color vision abnormalities. None of the patients were found to have blue-green color vision abnormalities. The classification of the color vision abnormality was based on the responses on AO HRR plates and the Farnsworth Munsell D15 arrangement test. Out of eight patients with color vision abnormality, three had deutan abnormality (deuteranomaly/deuteranopia), i.e., a prevalence of deutan of 0.80% in the general population, and 1.63% in males; three patients had protan abnormality (protanomaly/protanopia), i.e., a prevalence of 0.80% in the general population, and 1.63% in males; two patients had red-green color vision abnormality, which could not be classified further, i.e., a prevalence of 0.53% in the general population, and 1.08% in males. These two patients had red-green color vision abnormality on Ishihara pseudoisochromatic plates, and mild unclassified red-green color vision abnormality on AO HRR pseudoisochromatic plates. They arranged the colored discs of the Farnsworth Munsell D15 arrangement test in a pattern that did not match any axis of color vision abnormality. These patients need to be tested further with more refined tests like Farnsworth Munsell 100 Hue test or with an anomaloscope. Studies from Saudi Arabia, Central Africa, and Iraq have shown a higher prevalence of deutan abnormality (deuteranomaly and deuteranopia) compared to protan abnormality (protanomaly and protanopia), whereas our study revealed an equal prevalence of deutan and protan abnormalities [15].

In our study, three tests were employed in screening for color vision abnormality. None of the other studies in the literature has used more than two tests while testing for color vision abnormality. In our study, the classification of color vision abnormality was done on AO HRR pseudoisochromatic plates and the Farnsworth Munsell D15 arrangement test and grading of the defect was done on HRR pseudoisochromatic plates. Using three different tests to screen for color vision allowed us to compare the individual ability of each test to detect congenital color vision abnormality.

Ishihara pseudoisochromatic plates do not screen for tritan defect, whereas HRR pseudoisochromatic plates tests for tritan defect also. Tritan abnormalities are rare. Two out of eight patients with color vision abnormality could not arrange the colored discs of the Farnsworth Munsell test in a pattern that could be classified into any type of defect, i.e., 25% of patients with color vision abnormality had unreliable testing on the Farnsworth Munsell D15 arrangement test. These two patients had unclassified red-green color vision abnormality on Ishihara pseudoisochromatic plates and mild unclassified red-green color vision abnormality on AO HRR pseudoisochromatic plates test. Hence, the Farnsworth Munsell D15 arrangement test does not appear to identify mild color vision abnormality.

Ishihara appears to hold good for screening purposes. However, HRR pseudoisochromatic plates have the additional advantage of classifying and grading the abnormality, as well as screening for tritan abnormality. The Farnsworth Munsell D15 arrangement test can be used as an additional test, but not as the sole test in screening for color vision abnormality. Hence, we recommend that HRR pseudoisochromatic plates be used for screening for color vision abnormality.

Given their low prevalence, a sample size of 371 is small to detect abnormalities such as a blue-yellow color vision (tritan/tetaran) abnormality and color vision abnormality in females. Hence, we recommend studies with larger sample sizes to detect the prevalence of color vision abnormalities in the general population and females and to assess the prevalence of tritan abnormality.

Our study was performed in a tertiary eye care setting. Four patients, i.e., 50% of patients with color vision abnormality in our study, presented to us after being discovered to have defects on testing for various occupational purposes elsewhere, thereby introducing a selection bias. Hence, the prevalence reported in our study may be different from the prevalence in a community setting. In light of this, we recommend a larger community-based study using HRR pseudoisochromatic plates test to find out the true prevalence of color vision abnormality in a particular region.

## Conclusions

Congenital color vision abnormality cannot be treated. Therefore, early diagnosis as well as raising awareness of this condition are necessary to guide young people regarding their career options, given the fact that certain professions require normal color vision. Detection of color vision abnormality early in the life of an individual is very important to make informed decisions on future career options. Unfortunately, ophthalmologists often have the unpleasant task of informing applicants that they could not be given clearance for a particular employment because they have color vision abnormalities. Frustration and disappointment could be prevented if those affected could gain awareness of their condition earlier, which can help them in opting for other careers instead.

There is a need to raise awareness about color vision abnormality among the general population and enlighten affected individuals on alternative career options. Thus, we recommend that all children should undergo color vision screening before starting high school. Early detection of color vision abnormality in children by using Ishihara or AO HRR tests allows parents and teachers to make necessary adjustments to the teaching methods for appropriate learning.
